# Anaphylactoid reactions during implantation of polymer-filled ring stent grafts for treatment of abdominal aortic aneurysms

**DOI:** 10.1016/j.jvscit.2024.101551

**Published:** 2024-06-04

**Authors:** Félix H. Savoie-White, Caroline Marchand, Ievgen Gegiia, Yves Lachance-Lemay, Nathalie Gilbert, Julien Bernatchez, Ghislain Nourissat

**Affiliations:** a Division of Vascular Surgery, CHU de Québec, Québec, Canada; b Faculty of Medicine, Université Laval, Québec, Canada; c Division of Anesthesiology, CHU de Québec, Québec, Canada

**Keywords:** Abdominal aortic aneurysm, Anaphylactoid reaction, Endologix stent graft system, Endovascular surgery

## Abstract

Polymer ring stent grafts from Endologix are reliable to treat challenging abdominal aortic aneurysm anatomy (hostile neck and tortuous or narrow iliac arteries). Rare cases of anaphylactoid reactions have been reported during the filing time of the graft rings due to polymer leakage. Management with amines, an antihistamine drug, and supportive care quickly stabilized both of our patients, which permitted the continuation and completion of their surgery. In our experience, there was no death-related events nor negative impact on patients surgical and clinical outcomes. We report on polymer leakage using the Ovation IX and ALTO stent grafts resulting in an anaphylactoid reaction.

Since the first endovascular aneurysm repair (EVAR) by Parodi in 1990, several endograft proximal sealing technologies have been engineered.^1^ Design of the Endologix stent graft systems allow treatment of AAAs with challenging aortic neck anatomy that are outside the instructions for use (IFU) of other devices. These devices are of particular interest in patients with excessively angulated, short, or tapered proximal necks, and/or in patients with narrow, tortuous, or angulated iliac arteries.^2^^,^^3^ After suprarenal anchoring, the proximal landing site seal is achieved by filling two compliant and inflatable polymer rings with a low-viscosity radiopaque polymer that molds to the aortic wall. These inflatable rings have shown to reduce chronic radial forces while still achieving stable proximal neck anchoring up to 5 years.^2^^,^^3^ Polymer leaks are a unique and rare potential risk described in the early generations of polymer-filled stent grafts (Ovation and iX, both manufactured by TriVascular) but not yet described in the newer generation (ALTO, manufactured by Endologix).^4^

Anaphylactoid reaction during surgery requires emergent care. First signs may present as cardiovascular collapse or airway obstruction, associated or not with cutaneous manifestation.^5^^,^^6^ The management consists in the same as non-surgical anaphylactic shocks: adrenaline, antihistamine drugs, and support care.^7^ American Society of Anesthesiologists guidelines should be followed.

In addition to a literature review, we present two cases of anaphylactoid reaction observed during the filling of polymer rings. The goal is to shine a light on this potential complication during surgery and report on polymer leakage using the ALTO stent graft resulting in an anaphylactoid reaction. This case report has been reported in line with the Surgical CAse REport (SCARE) criteria.^8^ Written consent was received from both patients.

## Case report

### Case 1

A 68-year-old male with hypertension and hypercholesterolemia underwent EVAR for a 5.5 cm infra-renal AAA under general anesthesia. The patient had no previous history of allergies and an American Society of Anesthesiologists physical status of 2. An Ovation IX stent graft was chosen due to an angulated and short neck. During the polymer ring filling (which respected the instructions from manufacturer), the patients’ blood pressure dropped suddenly to 30 mmHg. Angiogram confirmed no aortic rupture but showed oozing of periprosthetic contrast polymer. The patient was started on an infusion of adrenaline and received 50 mg of diphenhydramine and a 1 L bolus of Ringer Lactate. The patient quickly stabilized with resolution of the hemodynamic status. Surgery was completed without any surgical complications. The patient spent 1 day in the intensive care unit with normal blood work. The patient recovered well without recidivism of hypotension and was discharged on postoperative day 3. The patient was well at the follow-up visit with adequate imaging.

### Case 2

A 67-year-old male with hypertension and hypercholesterolemia underwent EVAR for a 6.5-cm infra-renal AAA under general anesthesia. An ALTO stent graft was chosen outside of IFU due to short, angulated, and circumferential calcification of the neck. During the polymer ring filling (which respected the instructions from manufacturer), the patients’ blood pressure dropped to 80/30 mmHg. The patient was quickly stabilized with 25 mg of ephedrine, 50 mg of diphenhydramine, and a 1 L bolus of Ringer Lactate. Angiogram showed no rupture nor oozing of periprosthetic contrast polymer per inflation. However, both the vascular surgeon and the anesthesiologist concluded that the most likely diagnosis was anaphylactoid reaction due to the absence of other causes for transient hypotension (no previous history of adverse reaction to any medication used during anesthesia) and the timing of symptoms with polymer filling. The surgery was completed without complications. The patient stayed 1 day in the intensive care unit with normal blood work. The postoperative period was tainted with a positive test for SARS-CoV-2 and a type 1b endoleak that had to be managed by an endovascular approach. The patient was discharged on day 6 with no second episode of hypotension. The patient was well at the follow-up visit, which showed no complication.

## Discussion

The Ovation and ALTO stent grafts treat challenging aortic anatomy by filling two compliant and inflatable polymer rings with a low-viscosity radiopaque polymer that molds to the aortic wall.^2^^,^^3^ The polymer used is polyethylene glycol (PEG)-based. PEG is a synthetic, non-biodegradable polymer that is used widely in the medical field.^9^ Although PEG was thought to be non-immunogenic, evidence proved that, when conjugated with other materials, PEG may cause immunogenic responses.^10^

A literature review found three case reports of anaphylactoid reactions linked to polymer leakage during the implantation of an Ovation stent graft that have been reported.^11^, ^12^, ^13^ All cases have been summarized in the Table.

**Table:** A five-case summary of anaphylactoid reaction following the polymer filling of the Ovation stent graft platform

	Case 1	Case 2	Case 3	Case 4	Case 5
Authors	Savoie-White et al	Savoie-White et al	Sfyroeras et al	Siani et al	Simonte et al
Age, years	68	67	82	78	72
Sex	Male	Male	Male	Male	Male
AAA size, cm	5.5 asymptomatic	6.5 asymptomatic	5.3 asymptomatic	5.3 symptomatic	5.3 asymptomatic
ASA	2	2	3	3	3
Ovation generation	Second	Third	First	First	Second
Anesthesia type	General	General	Epidural	Local + sedation	Local + sedation
Signs and symptoms	Systemic hypotension	Systemic hypotension	Tingling sensation followed by cardiovascular arrest.	Back pain, loss of conscious, hypotension, and tachycardia.	Back pain followed by hypotension.
Anaphactic treatment	Epinephrine + volume repletion	Epinephrine + volume repletion	Intubation + epinephrine + volume repletion	Laryngeal mask, massive fluid therapy, and amines.	Intubation + epinephrine + volume repletion + aortic balloon
Follow-up	Patient was stabilized and surgery completed.	Patient was stabilized and surgery completed.	Surgery not completed due to hemodynamic instability. No further surgeries.	Patient was stabilized and surgery completed.	Patient was stabilized and surgery completed.
Adverse events	No adverse event	No adverse event	Unsuccessful surgical treatment	No adverse event	Extensive blisters and necrosis on the lower back and loss of leg strength

*ASA*, American Society of Anesthesiologists.

In 2019, Gupta et al reviewed 10,000 implanted Ovation devices from which 24 (0.2%) unpublished reports related to anaphylactoid reactions resulting during polymer filing were found. This review shows that polymer leak resulted in no mortality and only three non-excluded aneurysmal sacs. They conclude that possible reasons for augmented risk of polymer leak were initial manufacturing process, excessive graft manipulation during deployment, early ballooning prior to complete polymer cure, and lower body temperature. They also conclude that the Ovation stent graft platform is a safe and feasible technique to treat infrarenal AAAs.^14^ Unfortunately, no high-risk vascular anatomic features other than circumferential calcifications in patient 2 were identified to predict future risk of polymer leakage.

From 2018 to 2020, 12,763 Ovation iX systems were sold by Endologix and available for EVAR. Of those, 112 perioperative complications (0.9%) linked to polymer leaks were voluntarily reported to the United States Food and Drug Administration.^4^ The majority of these complications were hypotension but also included multi-organ failure, cardiac arrest, neurological complication, transient and prolonged hemodynamic instability, and spinal cord infarct.^4^

In 2020, Endologix modified their polymer-filled stent graft platform to incorporate design and manufacturing changes with the objective of eliminating areas of material weaknesses associated with polymer leaks. According to a literature review and the company, no polymer leaks have been reported with the ALTO stent graft. Although our experience is based on outside IFU, we report on a polymer leak that resulted in an anaphylactoid reaction using the ALTO stent graft.^4^ In our institution, more than 700 EVARs were implanted from 2015 to 2023, of which 90 stent grafts were from the Endologix platform. Our early perioperative complications using the inflatable polymer ring system are type IA (13%) and II endoleaks (14%), stent thrombosis or stenosis (14%), puncture site bleeding (6%), and anaphylactoid reaction (2%).

Lastly, we believe the new generation of polymer-filled stent graft systems are safe and that an assumed attitude is to systematically notify the anesthesiologist before polymer filling. Other recommendations for safe deployment of the Ovation stent graft system are found in the Fig.

**Fig:**
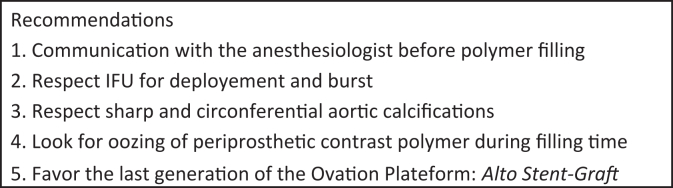
Recommendations to safely deploy the Ovation stent graft and detect early signs of polymer leak and systemic response. *IFU*, Instructions for use.

## Conclusion

In conclusion, anaphylactoid reaction is a rare complication while using polymer filled stent graft systems. The incidence of polymer leak is likely to change with the material weaknesses addressed but is nonetheless a factor that should be taken into consideration when choosing a personalized treatment plan to cure AAAs. In our experience, there were no death-related events nor negative impact on patients surgical and clinical outcomes. We strongly recommend systematically notifying the anesthesiologist before polymer filling to ensure adequate supervision. Further studies are needed to assess the evolution of this post-market risk with the ALTO stent graft.

## Disclosures

None.
